# Supplementary material for: Molecular clocks, biogeography and species diversity in Herichthys with evaluation of the role of Punta del Morro as a vicariant brake along the Mexican Transition Zone in the context of local and global time frame of cichlid diversification
Source: PeerJ. 2020 Apr 29;8:e8818. doi: 10.7717/peerj.8818 (PMC7195834; doi:10.7717/peerj.8818)
Supplement: Figure S2 — RAXML topology of the cytb Herichthys haplotype dataset including outgroup taxa. Node support values are from bootstrap analysis (only values above 70% are shown and only for putative species-level taxa and more inclusive nodes). [file peerj-08-8818-s004.pdf]

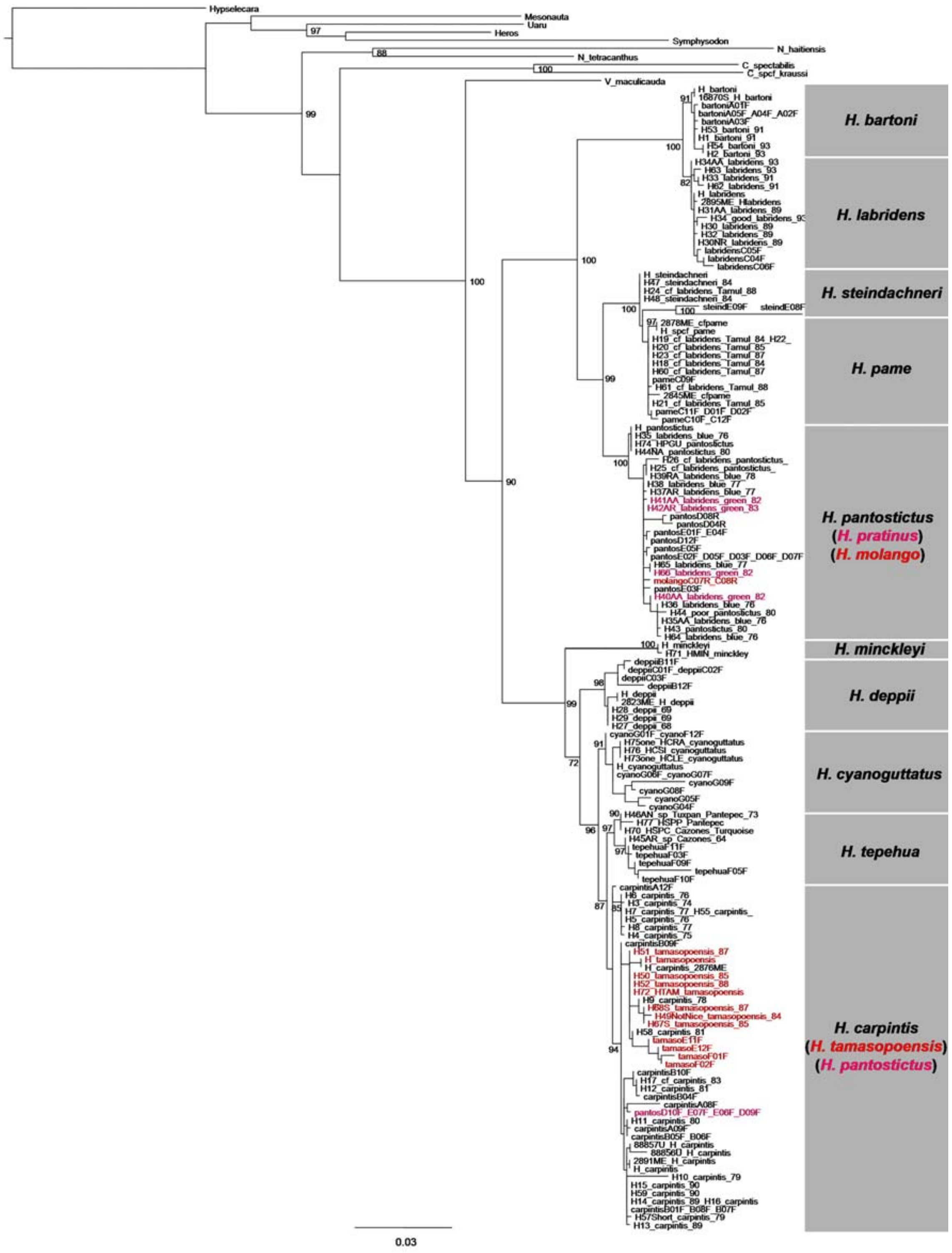

Supplemental material Fig. S2

RAXML topology of the cytb *Herichthys* haplotype dataset including outgroup taxa. Node support values are from bootstrap analysis (only values above 70% are shown and only for putative species-level taxa and more inclusive nodes).
