# Supplementary material for: Molecular clocks, biogeography and species diversity in Herichthys with evaluation of the role of Punta del Morro as a vicariant brake along the Mexican Transition Zone in the context of local and global time frame of cichlid diversification
Source: PeerJ. 2020 Apr 29;8:e8818. doi: 10.7717/peerj.8818 (PMC7195834; doi:10.7717/peerj.8818)
Supplement: Figure S3 — Microsoft Word - Supplementary material BEAST phylogeny of multilocus nDNA-mtDNA dataset of the Middle American and related Cichlidae (with outgroup taxa omitted) showing molecular clock date estimates and biogeographical reconstruction using Bayesian dispersal–vicariance (S-DIVA) analysis. The tree is from Rican et al. (2013). Rican et al. (2016) demonstrated that this dataset is strongly mtDNA dominated and identified conflicting nodes with a purely nDNA (ddRAD) phylogeny. Contrary to Rican et al. (2013), who used ichthyological provinces of Middle America as geographical units for the biogeographical analysis of the multilocus dataset, the present analysis used cichlid endemic areas (as the ddRAD analysis in Fig. 4). In the shown figure the reconstructed biogeography is simplified for easier orientation and hence the results of the detailed biogeographic analysis are reduced to the level of main units of Middle American geography and paleogeography. The biogeography at the level of these main units is used for the discussion (see Discussion) of the main constraints of paleogeography on Middle American cichlid diversification. The biogeographical analysis identifies the same set of extinctions (shown by dagger signs) as the ddRAD topology (Fig. 4), including those in the intervening area between Herichthys and its closest relatives (ancestrally from the Usumacinta river basin; in red). Based on the analysis separation of Herichthys from the remainder of its clade was thus not a vicariant event, but a dispersal event followed by isolation caused by extinctions. These extinctions (shown by the left pink column) are dated into a period of past high sea-level stands (14-11 Ma in this analysis; see Fig. 3) that occured during the Early-Middle Miocene (shown by the sea level curve from (Haq, Hardenbol & Vail, 1987)). A second and younger period of extinctions in northern Middle America (the right pink column) is also placed into a period of high sea levels in the Miocen [file peerj-08-8818-s005.pdf]

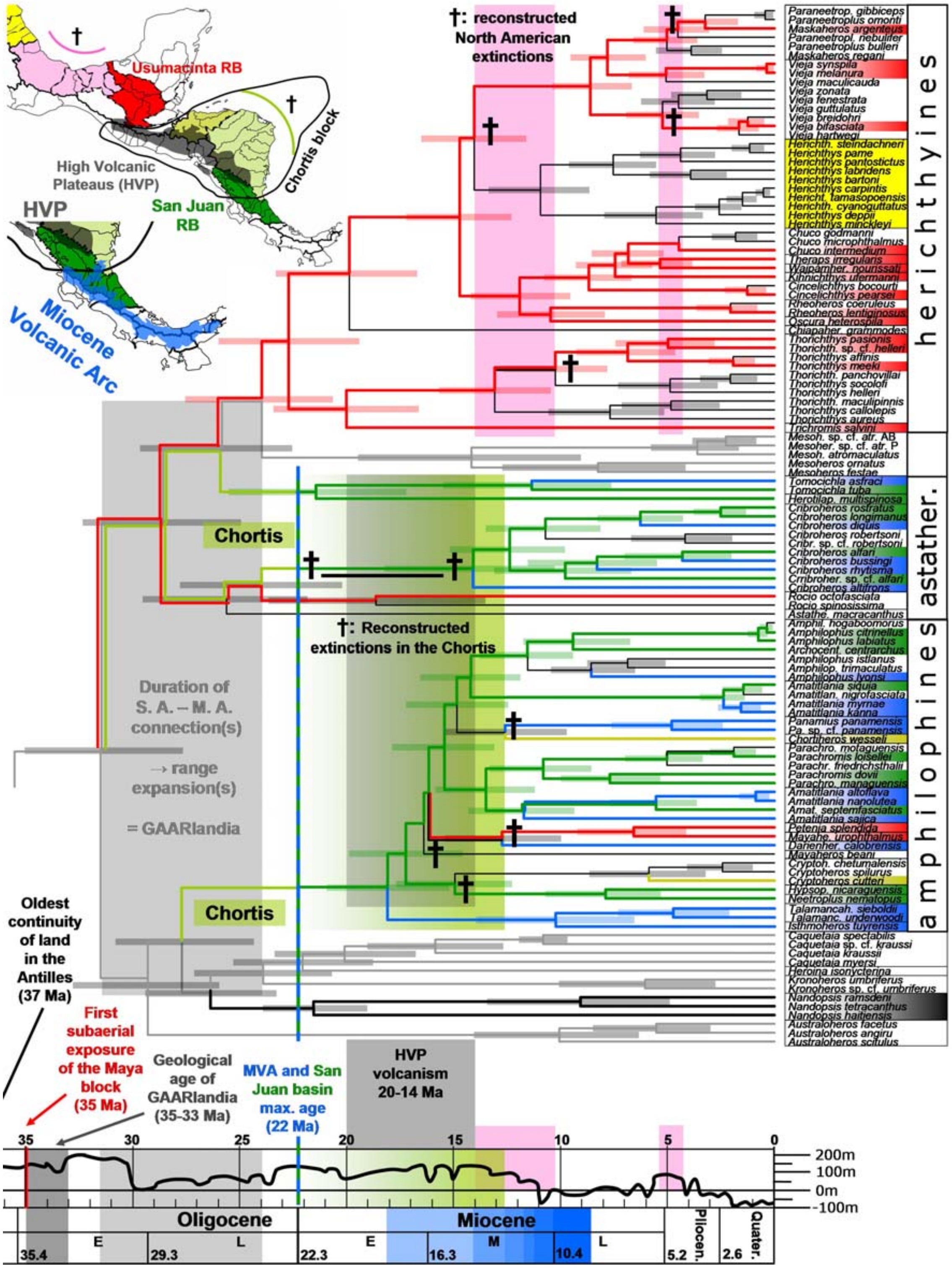

Supplemental material Fig. S3

BEAST phylogeny of multilocus nDNA-mtDNA dataset of the Middle American and related Cichlidae (with outgroup taxa omitted) showing molecular clock date estimates and biogeographical reconstruction using Bayesian dispersal–vicariance (S-DIVA) analysis. The tree is from Říčan et al. (2013). Říčan et al. (2016) demonstrated that this dataset is strongly mtDNA dominated and identified conflicting nodes with a purely nDNA (ddRAD) phylogeny.

Contrary to Říčan et al. (2013), who used ichthyological provinces of Middle America as geographical units for the biogeographical analysis of the multilocus dataset, the present analysis used cichlid endemic areas (as the ddRAD analysis in Fig. 4). In the shown figure the reconstructed biogeography is simplified for easier orientation and hence the results of the detailed biogeographic analysis are reduced to the level of main units of Middle American geography and paleogeography. The biogeography at the level of these main units is used for the discussion (see Discussion) of the main constraints of paleogeography on Middle American cichlid diversification.

The biogeographical analysis identifies the same set of extinctions (shown by dagger signs) as the ddRAD topology (Fig. 4), including those in the intervening area between *Herichthys* and its closest relatives (ancestrally from the Usumacinta river basin; in red). Based on the analysis separation of *Herichthys* from the remainder of its clade was thus not a vicariant event, but a dispersal event followed by isolation caused by extinctions. These extinctions (shown by the left pink column) are dated into a period of past high sea-level stands (14-11 Ma in this analysis; see Fig. 3) that occured during the Early-Middle Miocene (shown by the sea level curve from Haq et al., 1987). A second and younger period of extinctions in northern Middle America (the right pink column) is also placed into a period of high sea levels in the Miocene/Pliocene.

The main constraints of paleogeography on the Middle American cichlid diversification are as follows:

(I) The first constrain is the earliest possible colonization of the Antilles and Middle America. The oldest geological evidence for continuity in Caribbean land environments is from 37 Ma and for the Maya block from 35 Ma. This means that the oldest continuous survival of cichlids in the Greater Antilles (genus *Nandopsis* shown by black color in tree) and in Northern Middle America (the Maya block) cannot be older than 37 Ma and 35 Ma, respectively (indicated in the figure). Based on our biogeographical analyses (of this and the ddRAD dataset; Fig. 4) the communication between Middle America, the Antilles and South America (*Mesoheros* is the last South American node) occurred between 31.5 and 24 Ma (shown by light grey column). Our cichlid dating is thus ca 6 Ma younger than the maximum possible age and is thus compatible with the GAARlandia hypothesis.

(II) The second constrain is the maximum age of the San Juan basin, which formed when the Miocene Volcanic Arc (MVA) docked with the Chortis block of Nuclear Central America. The maximum age of the San Juan basin is based on geological literaure 22 Ma. The oldest date (22 Ma) for the formation of the San Juan basin is indicated by the vertical line at 22 Ma. Our cichlid biogeographical analyses reconstruct cichlid ancestral areas for both the astatheroine and amphilophine cichlids into the San Juan basin already at 31.5 and 27.5 Ma. While ancestral areas for the astatheroines and amphilophines include or are solely placed into the San Juan basin the clades are older than the San Juan basin and the only area of Central America that was subaerial previous to 22 Ma is the northern portion of the Chortis block. Based on the geological dating cichlids must have prior to 22 Ma lived on the Chortis block. The next two constraints explain this contradiction.

(III) The third constrain is the Ingimbrite volcanism that formed the High Volcanic Plateaus (HVP) of Nuclear Central America between 20 and 14 Ma. Our cichlid biogeographic analyses reconstruct several extinctions events that are found in this area of Central America. The dating of these extinction events agrees both with high sea levels during the Early-Middle Miocene as well as to the ingimbrite volcanism represented in the figure by the HVP. Only the HVP however explains the depauperate cichlid faunas of higher elevations in Nuclear Central America (Honduras, El Salvador, northern Nicaragua; Říčan et al., 2016). HVP volcanism is shown by the grey column (20-14 Ma) and the corresponding extinctions by the green column (start of the extinctions is uncertain do to the long branches at the base of the

clades but the extinctions are reconstructed as having occured between 22-13 Ma). The HVP explains why our biogeographical analyse place the basal nodes of the astatheroines and amphilophines into the San Juan basin, because all cichlid lineages have gone extinct in Chortis block during the HVP volcanism. The extinctions also probably explain the long basal branches in the astatheroines and especially the amphilophines. Our cichlid dating thus is at the maximum age to be compatible with the HVP constraint.

(IV) The fourth constrain is the Miocene Volcanic Arc (MVA) shown in blue and its emergence above seal levels is associated with the formation of the San Juan basin. Cichlid colonizations of the MVA thus could only have occured once this has become subaerial, starting at the earliest at 22 Ma. Or reconstructed colonizations of the MVA starts at 18 and are terminated at 8.6 Ma. Our cichlid dating is thus ca 4 Ma younger than the maximum possible age.

(V) The extinctions in cichlids in low-lying areas potentially influenced by the high sea levels of the Late Oligocene to Middle Miocene (23-14 (12) Ma) have a terminal age of 12.5 Ma for cichlids on the Chortis block and 10.5 on the Maya block. The cichlid datings can thus be at a maximum 1.5-3.5 Ma older. The extinctions in cichlids in low-lying areas of the Maya block potentially influenced by the high sea levels during the early Pliocene (5.5-4.5 Ma) are dated exactly into this time frame and thus the cichld dating at these nodes cannot the any older.
