# Supplementary material for: Molecular clocks, biogeography and species diversity in Herichthys with evaluation of the role of Punta del Morro as a vicariant brake along the Mexican Transition Zone in the context of local and global time frame of cichlid diversification
Source: PeerJ. 2020 Apr 29;8:e8818. doi: 10.7717/peerj.8818 (PMC7195834; doi:10.7717/peerj.8818)
Supplement: Supplemental Information 1 [file peerj-08-8818-s006.docx]

The new sequences generated in this study are available at GenBank under the accession numbers MK481080-MK481126

LOCUS       MK481080                1008 bp    DNA     linear   VRT 07-JUN-2019
DEFINITION  Herichthys carpintis haplotype B01F cytochrome b gene, partial cds;
            mitochondrial.
ACCESSION   MK481080
VERSION     MK481080
KEYWORDS    .
SOURCE      mitochondrion Herichthys carpintis (lowland cichlid)
  ORGANISM  Herichthys carpintis
            Eukaryota; Metazoa; Chordata; Craniata; Vertebrata; Euteleostomi;
            Actinopterygii; Neopterygii; Teleostei; Neoteleostei;
            Acanthomorphata; Ovalentaria; Cichlomorphae; Cichliformes;
            Cichlidae; New World cichlids; Cichlasomatinae; Heroini;
            Herichthys.
REFERENCE   1  (bases 1 to 1008)
  AUTHORS   Rican,O., Perez-Miranda,F., Lopez,B. and Mejia,O.
  TITLE     Molecular clocks, biogeography and species diversity in the genus
            Herichtys: Evaluating the role of Punta del Morro as a vicariant
            brake along the Mexican Transition Zone in the context of Middle
            American cichlid biogeography
  JOURNAL   Unpublished
REFERENCE   2  (bases 1 to 1008)
  AUTHORS   Rican,O., Perez-Miranda,F., Lopez,B. and Mejia,O.
  TITLE     Direct Submission
  JOURNAL   Submitted (01-FEB-2019) Zoologia, Instituto Politecnico
            Nacional-Escuela Nacional de Ciencias Biologicas, Carpio esq.Plan
            de Ayala s/n, Ciudad de Mexico, Mexico 11340, Mexico
COMMENT     ##Assembly-Data-START##
            Sequencing Technology :: Sanger dideoxy sequencing
            ##Assembly-Data-END##
FEATURES             Location/Qualifiers
     source          1..1008
                     /organism="Herichthys carpintis"
                     /organelle="mitochondrion"
                     /mol_type="genomic DNA"
                     /db_xref="taxon:131405"
                     /haplotype="B01F"
     CDS             <1..>1008
                     /codon_start=1
                     /transl_table=2
                     /product="cytochrome b"
                     /protein_id="QCY54433"
                     /translation="VWWNFGSLLGLCLAAQILTGLFLAMHYTSDIATAFSSVAHICRD
                     VNYGWLIRNMHANGASFFFICIYLHIGRGLYYGSYLYKETWNVGVVLLLLTMMTAFVG
                     YVLPWGQMSFWGATVITNLLSAIPYIGNSLVQWLWGGFSVDNATLTRFFAFHFLLPFI
                     IAAMTMIHLIFLHETGSTNPAGLNSDTDKISFHPYFSYKDLLGFAILLIALITLALFS
                     PNLLGDPDNFTPANPLVTPPHIKPEWYFLFAYAILRSIPNKLGGVLALLFSILILMLV
                     PILHTSKLRALTFRPLTQFLFWLLVADVIILTWIGGMPVEHPFIVIGQVASFLYFFIF
                     LI"
ORIGIN     
        1 gtttgatgaa acttcggctc cctactaggg ctctgtctcg ccgcccaaat tttaacaggc
       61 ctattccttg caatacacta cacttccgat atcgcaacag ccttctcatc cgttgcccac
      121 atctgccgag atgtaaatta tggctgacta atccgcaaca tacacgccaa cggcgcatct
      181 ttcttcttta tctgcattta ccttcacatc ggtcgaggac tatactacgg ctcttacctc
      241 tacaaagaaa catggaacgt cggcgttgtt ctcctcctct taacaataat aaccgcattc
      301 gtaggctacg tcctcccctg aggacaaata tccttttgag gtgccaccgt tatcaccaac
      361 cttctctccg caatccctta catcggcaac tccctagtcc aatgactctg aggaggcttt
      421 tcagtagaca atgccaccct cacccgattc tttgccttcc acttcctcct gccattcatc
      481 attgcagcca taacaataat tcacctaatc ttcctccacg aaaccggatc aacaaaccca
      541 gcaggcttaa actccgacac agacaaaatt tcattccacc cctacttctc ctacaaagat
      601 ctgctaggct ttgcaatcct acttatcgcc ctaatcacct tagccctctt ttcccctaac
      661 ctcctaggag acccagataa cttcaccccc gcaaaccccc tagtcacacc cccacatatc
      721 aaaccagaat gatacttcct atttgcttac gccatcctcc gatcaatccc caacaaacta
      781 ggaggtgtcc ttgcacttct tttctccatc ctaatcctca tactcgtccc aatcctccat
      841 acctcaaaac tccgagccct caccttccgg ccactcaccc aattcctatt ctggcttcta
      901 gttgcggacg tcattatctt aacttgaatc ggaggcatgc ctgttgaaca tccatttatc
      961 gtcatcggcc aagtcgcatc attcctctac ttctttattt tccttatc
//
LOCUS       MK481081                1008 bp    DNA     linear   VRT 07-JUN-2019
DEFINITION  Herichthys carpintis haplotype B05F cytochrome b gene, partial cds;
            mitochondrial.
ACCESSION   MK481081
VERSION     MK481081
KEYWORDS    .
SOURCE      mitochondrion Herichthys carpintis (lowland cichlid)
  ORGANISM  Herichthys carpintis
            Eukaryota; Metazoa; Chordata; Craniata; Vertebrata; Euteleostomi;
            Actinopterygii; Neopterygii; Teleostei; Neoteleostei;
            Acanthomorphata; Ovalentaria; Cichlomorphae; Cichliformes;
            Cichlidae; New World cichlids; Cichlasomatinae; Heroini;
            Herichthys.
REFERENCE   1  (bases 1 to 1008)
  AUTHORS   Rican,O., Perez-Miranda,F., Lopez,B. and Mejia,O.
  TITLE     Molecular clocks, biogeography and species diversity in the genus
            Herichtys: Evaluating the role of Punta del Morro as a vicariant
            brake along the Mexican Transition Zone in the context of Middle
            American cichlid biogeography
  JOURNAL   Unpublished
REFERENCE   2  (bases 1 to 1008)
  AUTHORS   Rican,O., Perez-Miranda,F., Lopez,B. and Mejia,O.
  TITLE     Direct Submission
  JOURNAL   Submitted (01-FEB-2019) Zoologia, Instituto Politecnico
            Nacional-Escuela Nacional de Ciencias Biologicas, Carpio esq.Plan
            de Ayala s/n, Ciudad de Mexico, Mexico 11340, Mexico
COMMENT     ##Assembly-Data-START##
            Sequencing Technology :: Sanger dideoxy sequencing
            ##Assembly-Data-END##
FEATURES             Location/Qualifiers
     source          1..1008
                     /organism="Herichthys carpintis"
                     /organelle="mitochondrion"
                     /mol_type="genomic DNA"
                     /db_xref="taxon:131405"
                     /haplotype="B05F"
     CDS             <1..>1008
                     /codon_start=1
                     /transl_table=2
                     /product="cytochrome b"
                     /protein_id="QCY54434"
                     /translation="VWWNFGSLLGLCLAAQILTGLFLAMHYTSDIATAFSSVAHICRD
                     VNYGWLIRNMHANGASFFFICIYLHIGRGLYYGSYLYKETWNVGVVLLLLTMMTAFVG
                     YVLPWGQMSFWGATVITNLLSAIPYIGNSLVQWLWGGFSVDNATLTRFFAFHFLLPFI
                     IAAMTMIHLIFLHETGSTNPAGLNSDTDKISFHPYFSYKDLLGFAILLIALITLALFS
                     PNLLGDPDNFTPANPLVTPPHIKPEWYFLFAYAILRSIPNKLGGVLALLFSILILMLV
                     PILHTSKLRALTFRPLTQFLFWLLVADVIILTWIGGMPVEHPFIVIGQVASFLYFFIF
                     LI"
ORIGIN     
        1 gtttgatgaa acttcggctc cctactaggg ctctgtctcg ccgcccaaat tttaacaggc
       61 ctattccttg caatacacta cacttccgat atcgcaacag ccttctcatc cgttgcccac
      121 atctgccgag atgtaaatta tggctgacta atccgcaaca tacacgccaa cggcgcatct
      181 ttcttcttta tctgcattta ccttcacatc ggtcgaggac tatactacgg ctcttacctc
      241 tacaaagaaa catggaacgt cggcgttgtt ctcctcctct taacaataat aaccgcattc
      301 gtaggctacg tcctcccctg aggacaaata tccttttgag gtgccaccgt tattaccaac
      361 cttctctccg caatccctta catcggcaac tccctagtcc aatgactctg aggaggcttt
      421 tcagtagaca atgccaccct cacccgattc tttgccttcc acttcctcct gccattcatc
      481 attgcagcca taacaataat tcacctaatc ttcctccacg aaaccggatc aacaaaccca
      541 gcaggcttaa actccgacac agacaaaatt tcattccacc cctacttctc ctacaaagat
      601 ctgctaggct ttgcaatcct acttatcgcc ctaatcacct tagccctctt ttcccctaac
      661 ctcctaggag acccagataa cttcaccccc gcaaaccccc tagtcacacc cccacatatc
      721 aaaccagaat gatacttcct atttgcttac gccatcctcc gatcaatccc caacaaacta
      781 ggaggtgtcc tcgcacttct tttctccatc ctaatcctca tactcgtccc aatcctccat
      841 acctcaaaac tccgagccct caccttccgg ccactcaccc aattcctatt ctggcttcta
      901 gttgcggacg tcattatctt aacttgaatc ggaggcatgc ctgttgaaca tccatttatc
      961 gtcatcggcc aagtcgcatc attcctctac ttctttattt tccttatc
//
LOCUS       MK481082                1008 bp    DNA     linear   VRT 07-JUN-2019
DEFINITION  Herichthys carpintis haplotype A09F cytochrome b gene, partial cds;
            mitochondrial.
ACCESSION   MK481082
VERSION     MK481082
KEYWORDS    .
SOURCE      mitochondrion Herichthys carpintis (lowland cichlid)
  ORGANISM  Herichthys carpintis
            Eukaryota; Metazoa; Chordata; Craniata; Vertebrata; Euteleostomi;
            Actinopterygii; Neopterygii; Teleostei; Neoteleostei;
            Acanthomorphata; Ovalentaria; Cichlomorphae; Cichliformes;
            Cichlidae; New World cichlids; Cichlasomatinae; Heroini;
            Herichthys.
REFERENCE   1  (bases 1 to 1008)
  AUTHORS   Rican,O., Perez-Miranda,F., Lopez,B. and Mejia,O.
  TITLE     Molecular clocks, biogeography and species diversity in the genus
            Herichtys: Evaluating the role of Punta del Morro as a vicariant
            brake along the Mexican Transition Zone in the context of Middle
            American cichlid biogeography
  JOURNAL   Unpublished
REFERENCE   2  (bases 1 to 1008)
  AUTHORS   Rican,O., Perez-Miranda,F., Lopez,B. and Mejia,O.
  TITLE     Direct Submission
  JOURNAL   Submitted (01-FEB-2019) Zoologia, Instituto Politecnico
            Nacional-Escuela Nacional de Ciencias Biologicas, Carpio esq.Plan
            de Ayala s/n, Ciudad de Mexico, Mexico 11340, Mexico
COMMENT     ##Assembly-Data-START##
            Sequencing Technology :: Sanger dideoxy sequencing
            ##Assembly-Data-END##
FEATURES             Location/Qualifiers
     source          1..1008
                     /organism="Herichthys carpintis"
                     /organelle="mitochondrion"
                     /mol_type="genomic DNA"
                     /db_xref="taxon:131405"
                     /haplotype="A09F"
     CDS             <1..>1008
                     /codon_start=1
                     /transl_table=2
                     /product="cytochrome b"
                     /protein_id="QCY54435"
                     /translation="VWWNFGSLLGLCLAAQILTGLFLAMHYTSDIATAFSSVAHICRD
                     VNYGWLIRNMHANGASFFFICIYLHIGRGLYYGSYLYKETWNVGVVLLLLTMMTAFVG
                     YVLPWGQMSFWGATVITNLLSAIPYIGNSLVQWLWGGFSVDNATLTRFFAFHFLLPFI
                     IAAMTMIHLIFLHETGSTNPAGLNSDTDKISFHPYFSYKDLLGFAILLIALITLALFS
                     PNLLGDPDNFTPANPLVTPPHIKPEWYFLFAYAILRSIPNKLGGVLALLFSILILMLV
                     PILHTSKLRALTFRPLTQFLFWLLVADVIILTWIGGMPVEHPFIVIGEVASFLYFFIF
                     LI"
ORIGIN     
        1 gtttgatgaa acttcggctc cctactaggg ctctgtctcg ccgcccaaat tttaacaggc
       61 ctattccttg caatacacta cacttccgat atcgcaacag ccttctcatc cgttgcccac
      121 atctgccgag atgtaaatta tggctgacta atccgcaaca tacacgccaa cggcgcatct
      181 ttcttcttta tctgcattta ccttcacatc ggtcgaggac tatactacgg ctcttacctc
      241 tacaaagaaa catggaacgt cggcgttgtt ctcctcctct taacaataat aaccgcattc
      301 gtaggctacg tcctcccctg aggacaaata tccttttgag gtgccaccgt tattaccaac
      361 cttctctccg caatccctta catcggcaac tccctagtcc aatgactctg aggaggcttt
      421 tcagtagaca atgccaccct cacccgattc tttgccttcc acttcctcct gccattcatc
      481 attgcagcca taacaataat tcacctaatc ttcctccacg aaaccggatc aacaaaccca
      541 gcaggcttaa actccgacac agacaaaatt tcattccacc cctacttctc ctacaaagat
      601 ctgctaggct ttgcaatcct acttatcgcc ctaatcacct tagccctctt ttcccctaac
      661 ctcctaggag acccagataa cttcaccccc gcaaaccccc tagtcacacc cccacatatc
      721 aaaccagaat gatacttcct atttgcttac gccatcctcc gatcaatccc caacaaacta
      781 ggaggtgtcc tcgcacttct tttctccatc ctaatcctca tactcgtccc aatcctccat
      841 acctcaaaac tccgagccct caccttccgg ccactcaccc aattcctatt ctggcttcta
      901 gttgcggacg tcattatctt aacttgaatc ggaggcatgc ctgttgaaca tccatttatc
      961 gtcatcggcg aagtcgcatc attcctctac ttctttattt tccttatc
//
LOCUS       MK481083                1008 bp    DNA     linear   VRT 07-JUN-2019
DEFINITION  Herichthys carpintis haplotype B04F cytochrome b gene, partial cds;
            mitochondrial.
ACCESSION   MK481083
VERSION     MK481083
KEYWORDS    .
SOURCE      mitochondrion Herichthys carpintis (lowland cichlid)
  ORGANISM  Herichthys carpintis
            Eukaryota; Metazoa; Chordata; Craniata; Vertebrata; Euteleostomi;
            Actinopterygii; Neopterygii; Teleostei; Neoteleostei;
            Acanthomorphata; Ovalentaria; Cichlomorphae; Cichliformes;
            Cichlidae; New World cichlids; Cichlasomatinae; Heroini;
            Herichthys.
REFERENCE   1  (bases 1 to 1008)
  AUTHORS   Rican,O., Perez-Miranda,F., Lopez,B. and Mejia,O.
  TITLE     Molecular clocks, biogeography and species diversity in the genus
            Herichtys: Evaluating the role of Punta del Morro as a vicariant
            brake along the Mexican Transition Zone in the context of Middle
            American cichlid biogeography
  JOURNAL   Unpublished
REFERENCE   2  (bases 1 to 1008)
  AUTHORS   Rican,O., Perez-Miranda,F., Lopez,B. and Mejia,O.
  TITLE     Direct Submission
  JOURNAL   Submitted (01-FEB-2019) Zoologia, Instituto Politecnico
            Nacional-Escuela Nacional de Ciencias Biologicas, Carpio esq.Plan
            de Ayala s/n, Ciudad de Mexico, Mexico 11340, Mexico
COMMENT     ##Assembly-Data-START##
            Sequencing Technology :: Sanger dideoxy sequencing
            ##Assembly-Data-END##
FEATURES             Location/Qualifiers
     source          1..1008
                     /organism="Herichthys carpintis"
                     /organelle="mitochondrion"
                     /mol_type="genomic DNA"
                     /db_xref="taxon:131405"
                     /haplotype="B04F"
     CDS             <1..>1008
                     /codon_start=1
                     /transl_table=2
                     /product="cytochrome b"
                     /protein_id="QCY54436"
                     /translation="VWWNFGSLLGLCLAAQILTGLFLAMHYTSDITTAFSSVAHICRD
                     VNYGWLIRNMHANGASFFFICIYLHIGRGLYYGSYLYKETWNVGVVLLLLTMMTAFVG
                     YVLPWGQMSFWGATVITNLLSAIPYIGNSLVQWLWGGFSVDNATLTRFFAFHFLLPFI
                     IAAMTMIHLIFLHETGSTNPAGLNSDTDKISFHPYFSYKDLLGFAILLIALITLALFS
                     PNLLGDPDNFTPANPLVTPPHIKPEWYFLFAYAILRSIPNKLGGVLALLFSILILMLV
                     PILHTSKLRALTFRPLTQFLFWLLVADVIILTWIGGMPVEHPFIVIGEVASFLYFFIF
                     LI"
ORIGIN     
        1 gtttgatgaa acttcggctc cctactaggg ctctgtctcg ccgcccaaat tttaacaggc
       61 ctattccttg caatacacta cacttccgat atcacgacag ccttctcatc cgttgcccac
      121 atctgccgag atgtaaatta tggctgacta atccgcaaca tacacgccaa cggcgcatct
      181 ttcttcttta tctgcattta ccttcacatc ggtcgaggac tatactacgg ctcttacctc
      241 tacaaagaaa catggaacgt cggcgttgtt ctcctcctct taacaataat aaccgcattc
      301 gtaggctacg tcctcccctg aggacaaata tccttttgag gtgccaccgt tatcaccaac
      361 cttctctccg caatccctta catcggcaac tccctagtcc aatgactctg aggaggcttt
      421 tcagtagaca atgccaccct cacccgattc tttgccttcc acttcctcct gccattcatc
      481 attgcagcca taacaataat ccacctaatc ttcctccacg aaaccggatc aacaaaccca
      541 gcaggcttaa actccgacac agacaaaatt tcattccacc cctacttctc ctacaaagat
      601 ctgctaggct ttgcaatcct acttatcgcc ctaatcacct tagccctctt ttcccctaac
      661 ctcctaggag acccagataa cttcaccccc gctaaccccc tagtcacacc cccacatatc
      721 aaaccagaat gatacttcct atttgcttac gccatcctcc gatcaatccc caacaaacta
      781 ggaggtgtcc tcgcacttct tttctccatc ctaatcctca tactcgtccc aatcctccat
      841 acctcaaaac tccgagccct caccttccgg ccactcaccc aattcctatt ctggcttcta
      901 gttgcggacg tcattatctt aacttgaatc ggaggcatgc ctgttgaaca tccatttatc
      961 gtcatcggcg aagtcgcatc attcctctac ttctttattt tccttatc
//
LOCUS       MK481084                1008 bp    DNA     linear   VRT 07-JUN-2019
DEFINITION  Herichthys carpintis haplotype B10F cytochrome b gene, partial cds;
            mitochondrial.
ACCESSION   MK481084
VERSION     MK481084
KEYWORDS    .
SOURCE      mitochondrion Herichthys carpintis (lowland cichlid)
  ORGANISM  Herichthys carpintis
            Eukaryota; Metazoa; Chordata; Craniata; Vertebrata; Euteleostomi;
            Actinopterygii; Neopterygii; Teleostei; Neoteleostei;
            Acanthomorphata; Ovalentaria; Cichlomorphae; Cichliformes;
            Cichlidae; New World cichlids; Cichlasomatinae; Heroini;
            Herichthys.
REFERENCE   1  (bases 1 to 1008)
  AUTHORS   Rican,O., Perez-Miranda,F., Lopez,B. and Mejia,O.
  TITLE     Molecular clocks, biogeography and species diversity in the genus
            Herichtys: Evaluating the role of Punta del Morro as a vicariant
            brake along the Mexican Transition Zone in the context of Middle
            American cichlid biogeography
  JOURNAL   Unpublished
REFERENCE   2  (bases 1 to 1008)
  AUTHORS   Rican,O., Perez-Miranda,F., Lopez,B. and Mejia,O.
  TITLE     Direct Submission
  JOURNAL   Submitted (01-FEB-2019) Zoologia, Instituto Politecnico
            Nacional-Escuela Nacional de Ciencias Biologicas, Carpio esq.Plan
            de Ayala s/n, Ciudad de Mexico, Mexico 11340, Mexico
COMMENT     ##Assembly-Data-START##
            Sequencing Technology :: Sanger dideoxy sequencing
            ##Assembly-Data-END##
FEATURES             Location/Qualifiers
     source          1..1008
                     /organism="Herichthys carpintis"
                     /organelle="mitochondrion"
                     /mol_type="genomic DNA"
                     /db_xref="taxon:131405"
                     /haplotype="B10F"
     CDS             <1..>1008
                     /codon_start=1
                     /transl_table=2
                     /product="cytochrome b"
                     /protein_id="QCY54437"
                     /translation="VWWNFGSLLGLCLAAQILTGLFLAMHYTSDIATAFSSVAHICRD
                     VNYGWLIRNMHANGASFFFICIYLHIGRGLYYGSYLYKETWNVGVVLLLLTMMTAFVG
                     YVLPWGQMSFWGATVITNLLSAIPYIGNSLVQWLWGGFSVDNATLTRFFAFHFLLPFI
                     IAAMTMIHLIFLHETGSTNPAGLNSDTDKISFHPYFSYKDLLGFAILLIALITLALFS
                     PNLLGDPDNFTPANPLVTPPHIKPEWYFLFAYAILRSIPNKLGGVLALLFSILILMLV
                     PILHTSKLRALTFRPLTQFLFWLLVADVIILTWIGGMPVEHPFIVIGQVASFLYFFIF
                     LI"
ORIGIN     
        1 gtttgatgaa acttcggctc cctactaggg ctctgtctcg ccgcccaaat tttaacaggc
       61 ctattccttg caatacacta cacttccgat atcgcgacag ccttctcatc cgttgcccac
      121 atctgccgag atgtaaatta tggctgacta atccgcaaca tacacgccaa cggcgcatct
      181 ttcttcttta tctgcattta ccttcacatc ggtcgaggac tatactacgg ctcttacctc
      241 tacaaagaaa catggaacgt cggcgttgtt ctcctcctct taacaataat aaccgcattc
      301 gtaggctacg tcctcccctg aggacaaata tccttttgag gtgccaccgt tatcaccaac
      361 cttctctccg caatccctta catcggcaac tccctagtcc aatgactctg aggaggcttt
      421 tcagtagaca atgccaccct cacccgattc tttgccttcc acttcctcct gccattcatc
      481 attgcagcca taacaataat ccacctaatc ttcctccacg aaaccggatc aacaaaccca
      541 gcaggcttaa actccgacac agacaaaatt tcattccacc cctacttctc ctacaaagat
      601 ctgctaggct ttgcaatcct acttatcgcc ctaatcacct tagccctctt ttcccctaac
      661 ctcctaggag acccagataa cttcaccccc gctaaccccc tagtcacacc cccacatatc
      721 aaaccagaat gatacttcct atttgcttac gccatcctcc gatcaatccc caacaaacta
      781 ggaggtgtcc tcgcacttct tttctccatc ctaatcctca tactcgtccc aatcctccat
      841 acctcaaaac tccgagccct caccttccgg ccactcaccc aattcctatt ctggcttcta
      901 gttgcggacg tcattatctt aacttgaatc ggaggcatgc ctgttgaaca tccatttatc
      961 gtcatcggcc aagtcgcatc attcctctac ttctttattt tccttatc
//
LOCUS       MK481085                1008 bp    DNA     linear   VRT 07-JUN-2019
DEFINITION  Herichthys pantostictus haplotype D10F cytochrome b gene, partial
            cds; mitochondrial.
ACCESSION   MK481085
VERSION     MK481085
KEYWORDS    .
SOURCE      mitochondrion Herichthys pantostictus
  ORGANISM  Herichthys pantostictus
            Eukaryota; Metazoa; Chordata; Craniata; Vertebrata; Euteleostomi;
            Actinopterygii; Neopterygii; Teleostei; Neoteleostei;
            Acanthomorphata; Ovalentaria; Cichlomorphae; Cichliformes;
            Cichlidae; New World cichlids; Cichlasomatinae; Heroini;
            Herichthys.
REFERENCE   1  (bases 1 to 1008)
  AUTHORS   Rican,O., Perez-Miranda,F., Lopez,B. and Mejia,O.
  TITLE     Molecular clocks, biogeography and species diversity in the genus
            Herichtys: Evaluating the role of Punta del Morro as a vicariant
            brake along the Mexican Transition Zone in the context of Middle
            American cichlid biogeography
  JOURNAL   Unpublished
REFERENCE   2  (bases 1 to 1008)
  AUTHORS   Rican,O., Perez-Miranda,F., Lopez,B. and Mejia,O.
  TITLE     Direct Submission
  JOURNAL   Submitted (01-FEB-2019) Zoologia, Instituto Politecnico
            Nacional-Escuela Nacional de Ciencias Biologicas, Carpio esq.Plan
            de Ayala s/n, Ciudad de Mexico, Mexico 11340, Mexico
COMMENT     ##Assembly-Data-START##
            Sequencing Technology :: Sanger dideoxy sequencing
            ##Assembly-Data-END##
FEATURES             Location/Qualifiers
     source          1..1008
                     /organism="Herichthys pantostictus"
                     /organelle="mitochondrion"
                     /mol_type="genomic DNA"
                     /db_xref="taxon:247398"
                     /haplotype="D10F"
     CDS             <1..>1008
                     /codon_start=1
                     /transl_table=2
                     /product="cytochrome b"
                     /protein_id="QCY54438"
                     /translation="VWWNFGSLLGLCLAAQILTGLFLAMHYTSDIATAFSSVAHICRD
                     VNYGWLIRNMHANGASFFFICIYLHIGRGLYYGSYLYKETWNVGVVLLLLTMMTAFVG
                     YVLPWGQMSFWGATVITNLLSAIPYIGNSLVQWLWGGFSVDNATLTRFFAFHFLLPFI
                     IAAMTMIHLIFLHETGSTNPAGLNSDTDKISFHPYFSYKDLLGFAILLIALITLALFS
                     PNLLGDPDNFTPANPLVTPPHIKPEWYFLFAYAILRSIPNKLGGVLALLFSILILMLV
                     PILHTSKLRALTFRPLTQFLFWLLVADVIILTWIGGMPVEHPFIVIGQVASFLYFFIF
                     LI"
ORIGIN     
        1 gtttgatgaa acttcggctc cctactaggg ctctgtctcg ccgcccaaat tttaacaggc
       61 ctattccttg caatacacta cacttccgat atcgcaacag ccttctcatc cgttgcccac
      121 atctgccgag atgtaaatta tggctgacta atccgcaaca tacacgccaa cggcgcatct
      181 ttcttcttta tctgcattta ccttcacatc ggtcgaggac tatactacgg ctcttacctc
      241 tacaaagaaa catgaaacgt cggcgttgtt ctcctcctct taacaataat aaccgcattc
      301 gtaggctacg tcctcccctg aggacaaata tccttttgag gtgccaccgt tatcaccaac
      361 cttctctccg caatccctta catcggcaac tccctagtcc aatgactctg aggaggcttt
      421 tcagtagaca atgccaccct cacccgattc tttgccttcc acttcctcct gccattcatc
      481 attgcagcca taacaataat tcacctaatc ttcctccacg aaaccggatc aacaaaccca
      541 gcaggcttaa actccgacac agacaaaatt tcattccacc cctacttctc ctacaaagat
      601 ctgctaggct ttgcaatcct acttatcgcc ctaatcacct tagccctctt ttcccctaac
      661 ctcctaggag acccagataa cttcaccccc gcaaaccccc tagtcacacc cccacatatc
      721 aaaccagaat gatacttcct atttgcttac gccatcctcc gatcaatccc caacaaacta
      781 ggaggtgtcc tcgcacttct tttctccatc ctaatcctca tactcgtccc aatcctccat
      841 acctcaaaac tccgggccct caccttccgg ccactcaccc aattcctatt ctggcttcta
      901 gttgcggacg tcattatctt aacttgaatc ggaggcatgc ctgttgaaca tccatttatc
      961 gtcatcggcc aagtcgcatc attcctctac ttctttattt tccttatc
//
LOCUS       MK481086                1008 bp    DNA     linear   VRT 07-JUN-2019
DEFINITION  Herichthys carpintis haplotype B09F cytochrome b gene, partial cds;
            mitochondrial.
ACCESSION   MK481086
VERSION     MK481086
KEYWORDS    .
SOURCE      mitochondrion Herichthys carpintis (lowland cichlid)
  ORGANISM  Herichthys carpintis
            Eukaryota; Metazoa; Chordata; Craniata; Vertebrata; Euteleostomi;
            Actinopterygii; Neopterygii; Teleostei; Neoteleostei;
            Acanthomorphata; Ovalentaria; Cichlomorphae; Cichliformes;
            Cichlidae; New World cichlids; Cichlasomatinae; Heroini;
            Herichthys.
REFERENCE   1  (bases 1 to 1008)
  AUTHORS   Rican,O., Perez-Miranda,F., Lopez,B. and Mejia,O.
  TITLE     Molecular clocks, biogeography and species diversity in the genus
            Herichtys: Evaluating the role of Punta del Morro as a vicariant
            brake along the Mexican Transition Zone in the context of Middle
            American cichlid biogeography
  JOURNAL   Unpublished
REFERENCE   2  (bases 1 to 1008)
  AUTHORS   Rican,O., Perez-Miranda,F., Lopez,B. and Mejia,O.
  TITLE     Direct Submission
  JOURNAL   Submitted (01-FEB-2019) Zoologia, Instituto Politecnico
            Nacional-Escuela Nacional de Ciencias Biologicas, Carpio esq.Plan
            de Ayala s/n, Ciudad de Mexico, Mexico 11340, Mexico
COMMENT     ##Assembly-Data-START##
            Sequencing Technology :: Sanger dideoxy sequencing
            ##Assembly-Data-END##
FEATURES             Location/Qualifiers
     source          1..1008
                     /organism="Herichthys carpintis"
                     /organelle="mitochondrion"
                     /mol_type="genomic DNA"
                     /db_xref="taxon:131405"
                     /haplotype="B09F"
     CDS             <1..>1008
                     /codon_start=1
                     /transl_table=2
                     /product="cytochrome b"
                     /protein_id="QCY54439"
                     /translation="VWWNFGSLLGLCLAAQILTGLFLAMHYTSDIATAFSSVAHICRD
                     VNYGWLIRNMHANGASFFFICIYLHIGRGLYYGSYLYKETWNVGVVLLLLTMMTAFVG
                     YVLPWGQMSFWGATVITNLLSAIPYIGNSLVQWLWGGFSVDNATLTRFFAFHFLLPFI
                     IAAMTMIHLIFLHETGSTNPAGLNSDTDKISFHPYFSYKDLLGFAILLIALITLALFS
                     PNLLGDPDNFTPANPLVTPPHIKPEWYFLFAYAILRSIPNKLGGVLALLFSILILMLV
                     PILHTSKLRALTFRPLTQFLFWLLVADVIILTWIGGMPVEHPFIVIGQVASFLYFFIF
                     LI"
ORIGIN     
        1 gtttgatgaa acttcggctc cctactaggg ctctgtctcg ccgcccaaat tttaacaggc
       61 ctattccttg caatacacta cacttccgat atcgcaacag ccttctcatc cgttgcccac
      121 atctgccgag atgtaaatta tggctgacta atccgcaaca tacacgccaa cggcgcatct
      181 ttcttcttta tctgcattta cctccacatc ggtcgaggac tatactacgg ctcttacctc
      241 tacaaagaaa catggaacgt cggcgttgtt ctcctcctct taacaataat aaccgcattc
      301 gtaggctacg tcctcccctg aggacaaata tccttttgag gtgccaccgt tatcaccaac
      361 cttctctccg caatccctta catcggcaac tccctagtcc aatgactctg aggaggcttt
      421 tcagtagaca atgccaccct cacccgattc tttgccttcc acttcctcct gccattcatc
      481 attgcagcca taacaataat tcacctaatc ttcctccacg aaaccggatc aacaaaccca
      541 gcaggcttaa actccgacac agacaaaatt tcattccacc cctacttctc ctacaaagat
      601 ctgctaggct ttgcaatcct acttatcgcc ctaatcacct tagccctctt ttcccctaac
      661 ctcctaggag acccagataa cttcaccccc gcaaaccccc tagtcacacc cccacatatc
      721 aaaccagaat gatacttcct atttgcttac gccatcctcc gatcaatccc caacaaacta
      781 ggaggtgtcc tcgcacttct tttctccatc ctaatcctca tactcgtccc aatcctccat
      841 acctcaaaac tccgagccct caccttccgg ccactcaccc aattcctatt ctggcttcta
      901 gttgcggacg tcattatctt aacttgaatc ggaggcatgc ctgttgaaca tccatttatc
      961 gtcatcggcc aagtcgcatc attcctctac ttctttattt tccttatc
//
LOCUS       MK481087                1008 bp    DNA     linear   VRT 07-JUN-2019
DEFINITION  Herichthys tamasopoensis haplotype F02F cytochrome b gene, partial
            cds; mitochondrial.
ACCESSION   MK481087
VERSION     MK481087
KEYWORDS    .
SOURCE      mitochondrion Herichthys tamasopoensis
  ORGANISM  Herichthys tamasopoensis
            Eukaryota; Metazoa; Chordata; Craniata; Vertebrata; Euteleostomi;
            Actinopterygii; Neopterygii; Teleostei; Neoteleostei;
            Acanthomorphata; Ovalentaria; Cichlomorphae; Cichliformes;
            Cichlidae; New World cichlids; Cichlasomatinae; Heroini;
            Herichthys.
REFERENCE   1  (bases 1 to 1008)
  AUTHORS   Rican,O., Perez-Miranda,F., Lopez,B. and Mejia,O.
  TITLE     Molecular clocks, biogeography and species diversity in the genus
            Herichtys: Evaluating the role of Punta del Morro as a vicariant
            brake along the Mexican Transition Zone in the context of Middle
            American cichlid biogeography
  JOURNAL   Unpublished
REFERENCE   2  (bases 1 to 1008)
  AUTHORS   Rican,O., Perez-Miranda,F., Lopez,B. and Mejia,O.
  TITLE     Direct Submission
  JOURNAL   Submitted (01-FEB-2019) Zoologia, Instituto Politecnico
            Nacional-Escuela Nacional de Ciencias Biologicas, Carpio esq.Plan
            de Ayala s/n, Ciudad de Mexico, Mexico 11340, Mexico
COMMENT     ##Assembly-Data-START##
            Sequencing Technology :: Sanger dideoxy sequencing
            ##Assembly-Data-END##
FEATURES             Location/Qualifiers
     source          1..1008
                     /organism="Herichthys tamasopoensis"
                     /organelle="mitochondrion"
                     /mol_type="genomic DNA"
                     /db_xref="taxon:247396"
                     /haplotype="F02F"
     CDS             <1..>1008
                     /codon_start=1
                     /transl_table=2
                     /product="cytochrome b"
                     /protein_id="QCY54440"
                     /translation="VWWNFGSLLGLCLAAQILTGLFLAMHYTSDIATAFSSVAHICRD
                     VNYGWLIRNMHANGASFFFICIYLHIGRGLYYGSYLYKETWNVGVVLLLLTMMTAFVG
                     YVLPWGQMSFWGATVITNLLSAIPYIGNSLVQWLWGGFSVDNATLTRFFAFHFLLPFI
                     IAAMTMIHLIFLHETGSTNPAGLNSDTDKISFHPYFSYKDLLGFAILLIALITLALFS
                     PNLLGDPDNFTPANPLVTPPHIKPEWYFLFAYAILRSIPNKLGGVLALLFSILILMLV
                     PILHTSKLRALTFRPLTQFLFWLLVADVIILTWIGGMPVEIPFIVIGQVASFLYFFIF
                     LI"
ORIGIN     
        1 gtttgatgaa acttcggctc cctactaggg ctctgtctcg ccgcccaaat tttaacaggc
       61 ctattccttg caatacacta cacttccgat atcgcaacag ccttctcatc cgttgcccac
      121 atctgccgag atgtaaatta tggctgacta atccgcaaca tacacgccaa cggcgcatct
      181 ttcttcttta tctgcattta ccttcacatc ggtcgaggac tatactacgg ctcttacctc
      241 tacaaagaaa catggaacgt cggcgttgtt ctcctcctct taacaataat aaccgcattc
      301 gtaggctacg tcctcccctg aggacaaata tccttttgag gtgccaccgt tatcaccaac
      361 cttctctccg caatccctta catcggcaac tccctagtcc aatgactctg aggaggcttt
      421 tcagtagaca atgccaccct cacccgattc tttgccttcc acttcctcct gccattcatc
      481 attgcagcca taacaataat ccacctaatc ttcctccacg aaaccggatc aacaaaccca
      541 gcaggcttaa actccgacac agacaaaatt tcattccacc cctacttctc ctacaaagat
      601 ctgctaggct ttgcaatcct acttatcgcc ctaatcacct tagccctctt ttcccctaac
      661 ctcctaggag acccagataa cttcaccccc gcaaaccccc tagtcacacc cccacatatt
      721 aaaccagaat gatacttcct atttgcttac gccatcctcc gatcaatccc caacaaacta
      781 ggaggtgtcc tcgcacttct tttctccatc ctaatcctca tactcgtccc aatcctccat
      841 acctcaaaac tccgagccct cactttccgg ccactcaccc aattcctatt ctggcttcta
      901 gttgcggacg tcattatctt aacttgaatc ggaggcatgc ctgttgaaat cccatttatc
      961 gtcatcggcc aggtcgcatc attcctctac ttctttattt tccttatc
//
LOCUS       MK481088                1008 bp    DNA     linear   VRT 07-JUN-2019
DEFINITION  Herichthys tamasopoensis haplotype E12F cytochrome b gene, partial
            cds; mitochondrial.
ACCESSION   MK481088
VERSION     MK481088
KEYWORDS    .
SOURCE      mitochondrion Herichthys tamasopoensis
  ORGANISM  Herichthys tamasopoensis
            Eukaryota; Metazoa; Chordata; Craniata; Vertebrata; Euteleostomi;
            Actinopterygii; Neopterygii; Teleostei; Neoteleostei;
            Acanthomorphata; Ovalentaria; Cichlomorphae; Cichliformes;
            Cichlidae; New World cichlids; Cichlasomatinae; Heroini;
            Herichthys.
REFERENCE   1  (bases 1 to 1008)
  AUTHORS   Rican,O., Perez-Miranda,F., Lopez,B. and Mejia,O.
  TITLE     Molecular clocks, biogeography and species diversity in the genus
            Herichtys: Evaluating the role of Punta del Morro as a vicariant
            brake along the Mexican Transition Zone in the context of Middle
            American cichlid biogeography
  JOURNAL   Unpublished
REFERENCE   2  (bases 1 to 1008)
  AUTHORS   Rican,O., Perez-Miranda,F., Lopez,B. and Mejia,O.
  TITLE     Direct Submission
  JOURNAL   Submitted (01-FEB-2019) Zoologia, Instituto Politecnico
            Nacional-Escuela Nacional de Ciencias Biologicas, Carpio esq.Plan
            de Ayala s/n, Ciudad de Mexico, Mexico 11340, Mexico
COMMENT     ##Assembly-Data-START##
            Sequencing Technology :: Sanger dideoxy sequencing
            ##Assembly-Data-END##
FEATURES             Location/Qualifiers
     source          1..1008
                     /organism="Herichthys tamasopoensis"
                     /organelle="mitochondrion"
                     /mol_type="genomic DNA"
                     /db_xref="taxon:247396"
                     /haplotype="E12F"
     CDS             <1..>1008
                     /codon_start=1
                     /transl_table=2
                     /product="cytochrome b"
                     /protein_id="QCY54441"
                     /translation="VWWNFGSLLGLCLAAQILTGLFLAMHYTSDIATAFSSVAHICRD
                     VNYGWLIRNMHANGASFFFICIYLHIGRGLYYGSYLYKETWNVGVVLLLLTMMTAFVG
                     YVLPWGQMSFWGATVITNLLSAIPYIGNSLVQWLWGGFSVDNATLTRFFAFHFLLPFI
                     IAAMTMIHLIFLHETGSTNPAGLNSDTDKISFHPYFSYKDLLGFAILLIALITLALFS
                     PNLLGDPDNFTPANPLVTPPHIKPEWYFLFAYAILRSIPNKLGGVLALLFSILILMLV
                     PILHTSKLRALTFRPLTQFLFWLLVADVIILTWIGGMPVEIQFIVIGQVASFLYFFIF
                     LI"
ORIGIN     
        1 gtttgatgaa acttcggctc cctactaggg ctctgtctcg ccgcccaaat tttaacaggc
       61 ctattccttg caatacacta cacttccgat atcgcaacag ccttctcatc cgttgcccac
      121 atctgccgag atgtaaatta tggctgacta atccgcaaca tacacgccaa cggcgcatct
      181 ttcttcttta tctgcattta ccttcacatc ggtcgaggac tatactacgg ctcttacctc
      241 tacaaagaaa catggaacgt cggcgttgtt ctcctcctct taacaataat aaccgcattc
      301 gtaggctacg tcctcccctg aggacaaata tccttttgag gtgccaccgt tatcaccaac
      361 cttctctccg caatccctta catcggcaac tccctagtcc aatgactctg aggaggcttt
      421 tcagtagaca atgccaccct cacccgattc tttgccttcc acttcctcct gccattcatc
      481 attgcagcca taacaataat ccacctaatc ttcctccacg aaaccggatc aacaaaccca
      541 gcaggcttaa actccgacac agacaaaatt tcattccacc cctacttctc ctacaaagat
      601 ctgctaggct ttgcaatcct acttatcgcc ctaatcacct tagccctctt ttcccctaac
      661 ctcctaggag acccagataa cttcaccccc gcaaaccccc tagtcacacc cccacatatt
      721 aaaccagaat gatacttcct atttgcttac gccatcctcc gatcaatccc caacaaacta
      781 ggaggtgtcc tcgcacttct tttctccatc ctaatcctca tactcgtccc aatcctccat
      841 acctcaaaac tccgagccct cactttccgg ccactcaccc aattcctatt ctggcttcta
      901 gttgcggacg tcattatctt aacttgaatc ggaggcatgc ctgttgaaat ccaatttatc
      961 gtcatcggcc aggtcgcatc attcctctac ttctttattt tccttatc
//
LOCUS       MK481089                1008 bp    DNA     linear   VRT 07-JUN-2019
DEFINITION  Herichthys tamasopoensis haplotype F01F cytochrome b gene, partial
            cds; mitochondrial.
ACCESSION   MK481089
VERSION     MK481089
KEYWORDS    .
SOURCE      mitochondrion Herichthys tamasopoensis
  ORGANISM  Herichthys tamasopoensis
            Eukaryota; Metazoa; Chordata; Craniata; Vertebrata; Euteleostomi;
            Actinopterygii; Neopterygii; Teleostei; Neoteleostei;
            Acanthomorphata; Ovalentaria; Cichlomorphae; Cichliformes;
            Cichlidae; New World cichlids; Cichlasomatinae; Heroini;
            Herichthys.
REFERENCE   1  (bases 1 to 1008)
  AUTHORS   Rican,O., Perez-Miranda,F., Lopez,B. and Mejia,O.
  TITLE     Molecular clocks, biogeography and species diversity in the genus
            Herichtys: Evaluating the role of Punta del Morro as a vicariant
            brake along the Mexican Transition Zone in the context of Middle
            American cichlid biogeography
  JOURNAL   Unpublished
REFERENCE   2  (bases 1 to 1008)
  AUTHORS   Rican,O., Perez-Miranda,F., Lopez,B. and Mejia,O.
  TITLE     Direct Submission
  JOURNAL   Submitted (01-FEB-2019) Zoologia, Instituto Politecnico
            Nacional-Escuela Nacional de Ciencias Biologicas, Carpio esq.Plan
            de Ayala s/n, Ciudad de Mexico, Mexico 11340, Mexico
COMMENT     ##Assembly-Data-START##
            Sequencing Technology :: Sanger dideoxy sequencing
            ##Assembly-Data-END##
FEATURES             Location/Qualifiers
     source          1..1008
                     /organism="Herichthys tamasopoensis"
                     /organelle="mitochondrion"
                     /mol_type="genomic DNA"
                     /db_xref="taxon:247396"
                     /haplotype="F01F"
     CDS             <1..>1008
                     /codon_start=1
                     /transl_table=2
                     /product="cytochrome b"
                     /protein_id="QCY54442"
                     /translation="VWWNFGSLLGLCLAAQILTGLFLAMHYTSDIATAFSSVAHICRD
                     VNYGWLIRNMHANGASFFFICIYLHIGRGLYYGSYLYKETWNVGVVLLLLTMMTAFVG
                     YVLPWGQMSFWGATVITNLLSAIPYIGNSLVQWLWGGFSVDNGTLTRFFAFHFLLAFI
                     IAAMTMIHLIFLHETGSTNPAGLNSDTDKISFHPYFSYKDLLGFAILLIALITLALFS
                     PNLLGDPDNFTPANPLVTPPHIKPEWYFLFAYAILRSIPNKLGGVLALLFSILILMLV
                     PILHTSKLRALTFRPLTQFLFWLLVADVIILTWIGGMPVVIPFIVIGQVASFLYFFIF
                     LI"
ORIGIN     
        1 gtttgatgaa acttcggctc cctactaggg ctctgtctcg ccgcccaaat tttaacaggc
       61 ctattccttg caatacacta cacttccgat atcgcaacag ccttctcatc cgttgcccac
      121 atctgccgag atgtaaatta tggctgacta atccgcaaca tacacgccaa cggcgcatct
      181 ttcttcttta tctgcattta ccttcacatc ggtcgaggac tatactacgg ctcttacctc
      241 tacaaagaaa catggaacgt cggcgttgtt ctcctcctct taacaataat aaccgcattc
      301 gtaggctacg tcctcccctg aggacaaata tccttttgag gtgccaccgt tatcaccaac
      361 cttctctccg caatccctta catcggcaac tccctagtcc aatgactctg aggaggcttt
      421 tcagtagaca atggcaccct cacccgattc tttgccttcc acttcctcct ggcattcatc
      481 attgcagcca taacaataat ccacctaatc ttcctccacg aaaccggatc aacaaaccca
      541 gcagggttaa actccgacac agacaaaatt tcattccacc cctacttctc ctacaaagat
      601 ctgctaggct ttgcaatcct acttatcgcc ctaatcacct tagccctctt ttcccctaac
      661 ctcctaggag acccagataa cttcaccccc gcaaaccccc tagtcacacc cccacatatt
      721 aaaccagaat gatacttcct atttgcttac gccatcctcc gatcaatccc caacaaacta
      781 ggaggtgtcc tcgcacttct tttctccatc ctaatcctca tactcgtccc aatcctccat
      841 acctcaaaac tccgagccct cactttccgg ccactcaccc aattcctatt ctggcttcta
      901 gttgcggacg tcattatctt aacttgaatc ggaggcatgc ctgttgtaat cccatttatc
      961 gtcatcggcc aggtcgcatc attcctctac ttctttattt tccttatc
//
LOCUS       MK481090                1008 bp    DNA     linear   VRT 07-JUN-2019
DEFINITION  Herichthys tamasopoensis haplotype E11F cytochrome b gene, partial
            cds; mitochondrial.
ACCESSION   MK481090
VERSION     MK481090
KEYWORDS    .
SOURCE      mitochondrion Herichthys tamasopoensis
  ORGANISM  Herichthys tamasopoensis
            Eukaryota; Metazoa; Chordata; Craniata; Vertebrata; Euteleostomi;
            Actinopterygii; Neopterygii; Teleostei; Neoteleostei;
            Acanthomorphata; Ovalentaria; Cichlomorphae; Cichliformes;
            Cichlidae; New World cichlids; Cichlasomatinae; Heroini;
            Herichthys.
REFERENCE   1  (bases 1 to 1008)
  AUTHORS   Rican,O., Perez-Miranda,F., Lopez,B. and Mejia,O.
  TITLE     Molecular clocks, biogeography and species diversity in the genus
            Herichtys: Evaluating the role of Punta del Morro as a vicariant
            brake along the Mexican Transition Zone in the context of Middle
            American cichlid biogeography
  JOURNAL   Unpublished
REFERENCE   2  (bases 1 to 1008)
  AUTHORS   Rican,O., Perez-Miranda,F., Lopez,B. and Mejia,O.
  TITLE     Direct Submission
  JOURNAL   Submitted (01-FEB-2019) Zoologia, Instituto Politecnico
            Nacional-Escuela Nacional de Ciencias Biologicas, Carpio esq.Plan
            de Ayala s/n, Ciudad de Mexico, Mexico 11340, Mexico
COMMENT     ##Assembly-Data-START##
            Sequencing Technology :: Sanger dideoxy sequencing
            ##Assembly-Data-END##
FEATURES             Location/Qualifiers
     source          1..1008
                     /organism="Herichthys tamasopoensis"
                     /organelle="mitochondrion"
                     /mol_type="genomic DNA"
                     /db_xref="taxon:247396"
                     /haplotype="E11F"
     CDS             <1..>1008
                     /codon_start=1
                     /transl_table=2
                     /product="cytochrome b"
                     /protein_id="QCY54443"
                     /translation="VWWNFGSLLGLCLAAQILTGLFLAMHYTSDIATAFSSVAHICRD
                     VNYGWLIRNMHANGASFFFICIYLHIGRGLYYGSYLYKETWNVGVVLLLLTMMTAFVG
                     YVLPWGQMSFWGATVITNLLSAIPYIGNSLVQWLWGGFSVDNATLTRFFAFHFLLPFI
                     IAAMTMIHLIFLHETGSTNPAGLNSDTDKISFHPYFSYKDLLGFAILLIALITLALFS
                     PNLLGDPDNFTPANPLVTPPHIKPEWYFLFAYAILRSIPNKLGGVLALLSSILILMLV
                     PILHTSKLRALTFRPLTQFLFWLLVADVIILTWIGGMPVEIQFIVIGQVASFLYFFIF
                     LI"
ORIGIN     
        1 gtttgatgaa acttcggctc cctactaggg ctctgtctcg ccgcccaaat tttaacaggc
       61 ctattccttg caatacacta cacttccgat atcgcaacag ccttctcatc cgttgcccac
      121 atctgccgag atgtaaatta tggctgacta atccgcaaca tacacgccaa cggcgcatct
      181 ttcttcttta tctgcattta cctccacatc ggtcgaggac tatactacgg ctcttacctc
      241 tacaaagaaa catggaacgt cggcgttgtt ctcctcctct taacaataat aaccgcattc
      301 gtaggctacg tcctcccctg aggacaaata tccttttgag gtgccaccgt tatcaccaac
      361 cttctctccg caatccctta catcggcaac tccctagtcc aatgactctg aggaggcttt
      421 tcagtagaca atgccaccct cacccgattc tttgccttcc acttcctcct gccattcatc
      481 attgcagcca taacaataat tcacctaatc ttcctccacg aaaccggatc aacaaaccca
      541 gcaggcttaa actccgacac agacaaaatt tcattccacc cctacttctc ctacaaagat
      601 ctgctaggct ttgcaatcct acttatcgcc ctaatcacct tagccctctt ttcccctaac
      661 ctcctaggag acccagataa cttcaccccc gcaaaccccc tagtcacacc cccacatatt
      721 aaaccagaat gatacttcct atttgcttac gccatcctcc gatcaatccc caacaaacta
      781 ggaggtgtcc tcgcacttct ttcctccatc ctaatcctca tactcgtccc aatcctccat
      841 acctcaaaac tccgagccct cactttccgg ccactcaccc aattcctatt ctggcttcta
      901 gttgcggacg tcattatctt aacttgaatc ggaggcatgc ctgttgaaat ccaatttatc
      961 gtcatcggcc aggtcgcatc attcctctac ttctttattt tccttatc
//
LOCUS       MK481091                1008 bp    DNA     linear   VRT 07-JUN-2019
DEFINITION  Herichthys tepehua haplotype F10F cytochrome b gene, partial cds;
            mitochondrial.
ACCESSION   MK481091
VERSION     MK481091
KEYWORDS    .
SOURCE      mitochondrion Herichthys tepehua
  ORGANISM  Herichthys tepehua
            Eukaryota; Metazoa; Chordata; Craniata; Vertebrata; Euteleostomi;
            Actinopterygii; Neopterygii; Teleostei; Neoteleostei;
            Acanthomorphata; Ovalentaria; Cichlomorphae; Cichliformes;
            Cichlidae; New World cichlids; Cichlasomatinae; Heroini;
            Herichthys.
REFERENCE   1  (bases 1 to 1008)
  AUTHORS   Rican,O., Perez-Miranda,F., Lopez,B. and Mejia,O.
  TITLE     Molecular clocks, biogeography and species diversity in the genus
            Herichtys: Evaluating the role of Punta del Morro as a vicariant
            brake along the Mexican Transition Zone in the context of Middle
            American cichlid biogeography
  JOURNAL   Unpublished
REFERENCE   2  (bases 1 to 1008)
  AUTHORS   Rican,O., Perez-Miranda,F., Lopez,B. and Mejia,O.
  TITLE     Direct Submission
  JOURNAL   Submitted (01-FEB-2019) Zoologia, Instituto Politecnico
            Nacional-Escuela Nacional de Ciencias Biologicas, Carpio esq.Plan
            de Ayala s/n, Ciudad de Mexico, Mexico 11340, Mexico
COMMENT     ##Assembly-Data-START##
            Sequencing Technology :: Sanger dideoxy sequencing
            ##Assembly-Data-END##
FEATURES             Location/Qualifiers
     source          1..1008
                     /organism="Herichthys tepehua"
                     /organelle="mitochondrion"
                     /mol_type="genomic DNA"
                     /db_xref="taxon:1830337"
                     /haplotype="F10F"
     CDS             <1..>1008
                     /codon_start=1
                     /transl_table=2
                     /product="cytochrome b"
                     /protein_id="QCY54444"
                     /translation="VWWNFGSLLGLCLAAQILTGLFLAMHYTSDIATAFSSVAHICRD
                     VNYGWLIRNMHANGASFFFICIYLHIGRGLYYGSYLYKETWNVGVILLLLTMMTAFVG
                     YVLPWGQMSFWGATVITNLLSAIPYIGNSLVQWLWGGFSVDNATLTRFFAFHFLLPFI
                     IAAMTMIHLIFLHETGSTNPAGLNSDTDKISFHPYFSYKDLLGFAILLIALITLALFS
                     PNLLGDPDNFTPANPLVTPPHIKPEWYFLFAYAILRSIPNKLGGVLALLFSILILMLV
                     PILHTSKLRALTFRPLTQFLFWLLVADVIILTWIGGMPVEHPFIVIGQVASFLYFFIF
                     LI"
ORIGIN     
        1 gtttgatgaa acttcggctc cctactaggg ctctgtctcg ccgcccaaat tttaacaggc
       61 ctattccttg caatacacta cacttccgat atcgcaacag ccttctcatc cgttgcccac
      121 atctgccgag atgtaaatta tggctgacta atccgcaaca tacacgccaa cggcgcatct
      181 ttcttcttta tctgcattta cctccacatc ggtcgaggac tgtactacgg ctcttacctc
      241 tacaaagaaa catggaacgt cggcgtcatt ctcctcctct taacaataat aaccgcattc
      301 gtaggctacg tcctcccctg aggacaaata tccttttgag gtgccaccgt tatcaccaac
      361 cttctctccg caatccctta catcggcaac tccctagtcc aatgactctg aggaggcttt
      421 tcagtagaca atgccaccct cacccgattc tttgccttcc acttcctcct gccattcatc
      481 attgcagcca taacaataat tcacctaatc ttcctccacg aaaccggatc aacaaaccca
      541 gcaggcttaa actccgacac agacaaaatt tcattccacc cctacttttc ctacaaagat
      601 ctgctaggct ttgccatcct acttatcgcc ctaatcacct tagccctctt ttcccccaat
      661 ctcctaggag acccagacaa cttcaccccc gcaaaccccc tagtcacacc cccacatatc
      721 aaaccagaat gatacttcct atttgcttac gccatcctcc gatcaatccc caacaaacta
      781 gggggggtgc tcgcacttct tttctccatc ctgatcctca tactcgtccc aatcctccat
      841 acctcaaaac tccgagccct taccttccgg ccactcaccc aattcctatt ctggcttcta
      901 gttgcggacg tcattatctt aacttgaatc ggaggcatgc ctgttgaaca tccatttatc
      961 gtcatcggcc aagtcgcatc attcctctac ttctttatct tccttatc
//
LOCUS       MK481092                1008 bp    DNA     linear   VRT 07-JUN-2019
DEFINITION  Herichthys tepehua haplotype F05F cytochrome b gene, partial cds;
            mitochondrial.
ACCESSION   MK481092
VERSION     MK481092
KEYWORDS    .
SOURCE      mitochondrion Herichthys tepehua
  ORGANISM  Herichthys tepehua
            Eukaryota; Metazoa; Chordata; Craniata; Vertebrata; Euteleostomi;
            Actinopterygii; Neopterygii; Teleostei; Neoteleostei;
            Acanthomorphata; Ovalentaria; Cichlomorphae; Cichliformes;
            Cichlidae; New World cichlids; Cichlasomatinae; Heroini;
            Herichthys.
REFERENCE   1  (bases 1 to 1008)
  AUTHORS   Rican,O., Perez-Miranda,F., Lopez,B. and Mejia,O.
  TITLE     Molecular clocks, biogeography and species diversity in the genus
            Herichtys: Evaluating the role of Punta del Morro as a vicariant
            brake along the Mexican Transition Zone in the context of Middle
            American cichlid biogeography
  JOURNAL   Unpublished
REFERENCE   2  (bases 1 to 1008)
  AUTHORS   Rican,O., Perez-Miranda,F., Lopez,B. and Mejia,O.
  TITLE     Direct Submission
  JOURNAL   Submitted (01-FEB-2019) Zoologia, Instituto Politecnico
            Nacional-Escuela Nacional de Ciencias Biologicas, Carpio esq.Plan
            de Ayala s/n, Ciudad de Mexico, Mexico 11340, Mexico
COMMENT     ##Assembly-Data-START##
            Sequencing Technology :: Sanger dideoxy sequencing
            ##Assembly-Data-END##
FEATURES             Location/Qualifiers
     source          1..1008
                     /organism="Herichthys tepehua"
                     /organelle="mitochondrion"
                     /mol_type="genomic DNA"
                     /db_xref="taxon:1830337"
                     /haplotype="F05F"
     CDS             <1..>1008
                     /codon_start=1
                     /transl_table=2
                     /product="cytochrome b"
                     /protein_id="QCY54445"
                     /translation="VWWNFGSLLGLCLAAQILTGLFLAMHYTSDIATAFSSVAHICRD
                     VNYGWLIRNMHANGASFFFICIYLHIGRGLYYGSYLYKETWNVGVILLLLTMMTAFVG
                     YVLPWGQMSFWGATGITNLLSAIPYIGNSLVQWLWGGFSVDNGTLTRFFAFHFLLPFI
                     IGAMTMIHLIFLHETGSTNPAGLNSDTDKISFHPYFSYKDLLGFAILLIALITLALFS
                     PNLLGDPDNFTPANPLVTPPHIKPEWYFLFAYAILRSIPNKLGGVLPILFSILNLILV
                     PILHTSKLRALTFPPLTQFLFWLLFADVIILTWIGGVPVEHPFIVIGEVGSFLYFFIF
                     LI"
ORIGIN     
        1 gtttgatgaa acttcggctc cctactaggg ctctgtctcg ccgcccaaat tttaacaggc
       61 ctattccttg caatacacta cacttccgat atcgcaacag ccttctcatc cgttgcccac
      121 atctgccgag atgtaaatta tggctgacta atccgcaaca tacacgccaa cggcgcatct
      181 ttcttcttta tctgcattta cctccacatc ggtcgaggac tgtactacgg ctcttacctc
      241 tacaaagaaa catggaacgt cggcgtcatt ctcctcctct taacaataat aaccgcattc
      301 gtaggctacg tcctcccctg aggacaaata tccttttgag gtgccaccgg tatcaccaac
      361 cttctctccg caatccctta catcggcaac tccctagtcc aatgactctg aggaggcttt
      421 tcagtagaca atggcaccct cacccgattc tttgccttcc acttcctcct gccattcatc
      481 attggagcca taacaataat tcacctaatc ttcctccacg aaaccggatc aacaaaccca
      541 gcaggcttaa actccgacac agacaaaatt tcattccacc cctacttttc ctacaaagat
      601 ctactaggct ttgccatcct acttatcgcc ctaatcacct tagccctctt ttcccccaat
      661 ctcctaggag acccagacaa cttcaccccc gcaaaccccc tagtcacacc cccacatatc
      721 aaaccagaat gatacttcct atttgcttac gccatcctcc gatcaatccc caacaaactg
      781 gggggggtgc tcccaattct tttctccatc ctgaacctca tccttgtccc aatcctccat
      841 acctcaaaac tccgagccct taccttcccg ccactcaccc aattcctatt ctggcttctt
      901 tttgcggacg tcattatctt aacttgaatc ggaggcgtgc ctgttgaaca tccatttatc
      961 gtcatcggcg aagtcggatc attcctctac ttctttatct tccttatc
//
LOCUS       MK481093                1008 bp    DNA     linear   VRT 07-JUN-2019
DEFINITION  Herichthys tepehua haplotype F09F cytochrome b gene, partial cds;
            mitochondrial.
ACCESSION   MK481093
VERSION     MK481093
KEYWORDS    .
SOURCE      mitochondrion Herichthys tepehua
  ORGANISM  Herichthys tepehua
            Eukaryota; Metazoa; Chordata; Craniata; Vertebrata; Euteleostomi;
            Actinopterygii; Neopterygii; Teleostei; Neoteleostei;
            Acanthomorphata; Ovalentaria; Cichlomorphae; Cichliformes;
            Cichlidae; New World cichlids; Cichlasomatinae; Heroini;
            Herichthys.
REFERENCE   1  (bases 1 to 1008)
  AUTHORS   Rican,O., Perez-Miranda,F., Lopez,B. and Mejia,O.
  TITLE     Molecular clocks, biogeography and species diversity in the genus
            Herichtys: Evaluating the role of Punta del Morro as a vicariant
            brake along the Mexican Transition Zone in the context of Middle
            American cichlid biogeography
  JOURNAL   Unpublished
REFERENCE   2  (bases 1 to 1008)
  AUTHORS   Rican,O., Perez-Miranda,F., Lopez,B. and Mejia,O.
  TITLE     Direct Submission
  JOURNAL   Submitted (01-FEB-2019) Zoologia, Instituto Politecnico
            Nacional-Escuela Nacional de Ciencias Biologicas, Carpio esq.Plan
            de Ayala s/n, Ciudad de Mexico, Mexico 11340, Mexico
COMMENT     ##Assembly-Data-START##
            Sequencing Technology :: Sanger dideoxy sequencing
            ##Assembly-Data-END##
FEATURES             Location/Qualifiers
     source          1..1008
                     /organism="Herichthys tepehua"
                     /organelle="mitochondrion"
                     /mol_type="genomic DNA"
                     /db_xref="taxon:1830337"
                     /haplotype="F09F"
     CDS             <1..>1008
                     /codon_start=1
                     /transl_table=2
                     /product="cytochrome b"
                     /protein_id="QCY54446"
                     /translation="VWWNFGSLLGLCLAAQILTGLFLAMHYTSDIATAFSSVAHICRD
                     VNYGWLIRNMHANGASFFFICIYLHIGRGLYYGSYLYKETWNVGVILLLLTMMTAFVG
                     YVLPWGQMSFWGATVITNLLSAIPYIGNSLVQWLWGGFSVDNATLTRFFAFHFLLPFI
                     IAAMTMIHLIFLHETGSTNPAGLNSDTDKISFHPYFSYKDLLGFAILLIALITLALFS
                     PNLLGDPDNFTPANPLVTPPHIKPEWYFLFAYAILRSIPNKLGGVLALLFSILILMLV
                     PILHTSKLRALTFRPLTQFLFWLLVADVIILTWIGGMPVEHPFIVIGQVASFLYFFIF
                     LI"
ORIGIN     
        1 gtttgatgaa acttcggctc cctactaggg ctctgtctcg ccgcccaaat tttaacaggc
       61 ctattccttg caatacacta cacttccgat atcgcaacag ccttctcatc cgttgcccac
      121 atctgccgag atgtaaatta tggctgacta atccgcaaca tacacgccaa cggcgcatct
      181 ttcttcttta tctgcattta cctccacatc ggtcgaggac tgtactacgg ctcttacctc
      241 tacaaagaaa catggaacgt cggcgtcatt ctcctcctct taacaataat aaccgcattc
      301 gtaggctacg tcctcccctg aggacaaata tccttttgag gtgccaccgt tatcaccaac
      361 cttctctccg caatccctta catcggcaac tccctagtcc aatgactctg aggaggcttt
      421 tcagtagaca atgccaccct cacccgattc tttgccttcc acttcctcct gccattcatc
      481 attgcagcca taacaataat tcacctaatc ttcctccacg aaaccggatc aacaaaccca
      541 gcaggcttaa actccgacac agacaaaatt tcattccacc cctacttttc ctacaaagat
      601 ctgctaggct ttgccatcct acttatcgcc ctaatcacct tagccctctt ttcccccaat
      661 ctcctaggag acccagacaa cttcaccccc gcaaaccccc tagtcacacc cccacatatc
      721 aaaccagaat gatacttcct atttgcttac gccatcctcc gatcaatccc caacaaacta
      781 gggggtgtgc tcgcacttct tttctccatc ctgatcctca tactcgtccc aatcctccat
      841 acctcaaaac tccgagccct taccttccgg ccactcaccc aattcctatt ctggcttcta
      901 gttgcggacg tcattatctt aacttgaatc ggaggcatgc ctgttgaaca tccatttatc
      961 gtcatcggcc aagtcgcatc attcctctac ttctttatct tccttatc
//
LOCUS       MK481094                1008 bp    DNA     linear   VRT 07-JUN-2019
DEFINITION  Herichthys tepehua haplotype F11F cytochrome b gene, partial cds;
            mitochondrial.
ACCESSION   MK481094
VERSION     MK481094
KEYWORDS    .
SOURCE      mitochondrion Herichthys tepehua
  ORGANISM  Herichthys tepehua
            Eukaryota; Metazoa; Chordata; Craniata; Vertebrata; Euteleostomi;
            Actinopterygii; Neopterygii; Teleostei; Neoteleostei;
            Acanthomorphata; Ovalentaria; Cichlomorphae; Cichliformes;
            Cichlidae; New World cichlids; Cichlasomatinae; Heroini;
            Herichthys.
REFERENCE   1  (bases 1 to 1008)
  AUTHORS   Rican,O., Perez-Miranda,F., Lopez,B. and Mejia,O.
  TITLE     Molecular clocks, biogeography and species diversity in the genus
            Herichtys: Evaluating the role of Punta del Morro as a vicariant
            brake along the Mexican Transition Zone in the context of Middle
            American cichlid biogeography
  JOURNAL   Unpublished
REFERENCE   2  (bases 1 to 1008)
  AUTHORS   Rican,O., Perez-Miranda,F., Lopez,B. and Mejia,O.
  TITLE     Direct Submission
  JOURNAL   Submitted (01-FEB-2019) Zoologia, Instituto Politecnico
            Nacional-Escuela Nacional de Ciencias Biologicas, Carpio esq.Plan
            de Ayala s/n, Ciudad de Mexico, Mexico 11340, Mexico
COMMENT     ##Assembly-Data-START##
            Sequencing Technology :: Sanger dideoxy sequencing
            ##Assembly-Data-END##
FEATURES             Location/Qualifiers
     source          1..1008
                     /organism="Herichthys tepehua"
                     /organelle="mitochondrion"
                     /mol_type="genomic DNA"
                     /db_xref="taxon:1830337"
                     /haplotype="F11F"
     CDS             <1..>1008
                     /codon_start=1
                     /transl_table=2
                     /product="cytochrome b"
                     /protein_id="QCY54447"
                     /translation="VWWNFGSLLGLCLAAQILTGLFLAMHYTSDIATAFSSVAHICRD
                     VNYGWLIRNMHANGASFFFICIYLHIGRGLYYGSYLYKETWNVGVILLLLTMMTAFVG
                     YVLPWGQMSFWGATVITNLLSAIPYIGNSLVQWLWGGFSVDNATLTRFFAFHFLLPFI
                     IAAMTMIHLIFLHETGSTNPAGLNSDTDKISFHPYFSYKDLLGFAILLIALITLALFS
                     PNLLGDPDNFTPANPLVTPPHIKPEWYFLFAYAILRSIPNKLGGVLALLFSILILMLV
                     PILHTSKLRALTFRPLTQFLFWLLVADVIILTWIGGMPVEHPFIVIGQVASFLYFFIF
                     LI"
ORIGIN     
        1 gtttgatgaa acttcggctc cctactaggg ctctgtctcg ccgcccaaat tttaacaggc
       61 ctattccttg caatacacta cacttccgat atcgcaacag ccttctcatc cgttgcccac
      121 atctgccgag atgtaaatta tggctgacta atccgcaaca tacacgccaa cggcgcatct
      181 ttcttcttta tctgcattta cctccacatc ggtcgaggac tgtactacgg ctcttacctc
      241 tacaaagaaa catggaacgt cggcgtcatt ctcctcctct taacaataat aaccgcattc
      301 gtaggctacg tcctcccctg aggacaaata tccttttgag gtgccaccgt tatcaccaac
      361 cttctctccg caatccctta catcggcaac tccctagtcc aatgactctg aggaggcttt
      421 tcagtagaca atgccaccct cacccgattc tttgccttcc acttcctcct gccattcatc
      481 attgcagcca taacaataat tcacctaatc ttcctccacg aaaccggatc aacaaaccca
      541 gcaggcttaa actccgacac agacaaaatt tcattccacc cctacttttc ctacaaagat
      601 ctgctaggct ttgccatcct acttatcgcc ctaatcacct tagccctctt ttcccccaat
      661 ctcctaggag acccagacaa cttcaccccc gcaaaccccc tagtcacacc cccacatatc
      721 aaaccagaat gatacttcct atttgcttac gccatcctcc gatcaatccc caacaaacta
      781 ggaggtgtcc tcgcacttct tttctccatc ctgatcctca tactcgtccc aatcctccat
      841 acctcaaaac tccgagccct taccttccgg ccactcaccc aattcctatt ctggcttcta
      901 gttgcggacg tcattatctt aacttgaatc ggaggcatgc ctgttgaaca tccatttatc
      961 gtcatcggcc aagtcgcatc attcctctac ttctttatct tccttatc
//
LOCUS       MK481095                1008 bp    DNA     linear   VRT 07-JUN-2019
DEFINITION  Herichthys tepehua haplotype F03F cytochrome b gene, partial cds;
            mitochondrial.
ACCESSION   MK481095
VERSION     MK481095
KEYWORDS    .
SOURCE      mitochondrion Herichthys tepehua
  ORGANISM  Herichthys tepehua
            Eukaryota; Metazoa; Chordata; Craniata; Vertebrata; Euteleostomi;
            Actinopterygii; Neopterygii; Teleostei; Neoteleostei;
            Acanthomorphata; Ovalentaria; Cichlomorphae; Cichliformes;
            Cichlidae; New World cichlids; Cichlasomatinae; Heroini;
            Herichthys.
REFERENCE   1  (bases 1 to 1008)
  AUTHORS   Rican,O., Perez-Miranda,F., Lopez,B. and Mejia,O.
  TITLE     Molecular clocks, biogeography and species diversity in the genus
            Herichtys: Evaluating the role of Punta del Morro as a vicariant
            brake along the Mexican Transition Zone in the context of Middle
            American cichlid biogeography
  JOURNAL   Unpublished
REFERENCE   2  (bases 1 to 1008)
  AUTHORS   Rican,O., Perez-Miranda,F., Lopez,B. and Mejia,O.
  TITLE     Direct Submission
  JOURNAL   Submitted (01-FEB-2019) Zoologia, Instituto Politecnico
            Nacional-Escuela Nacional de Ciencias Biologicas, Carpio esq.Plan
            de Ayala s/n, Ciudad de Mexico, Mexico 11340, Mexico
COMMENT     ##Assembly-Data-START##
            Sequencing Technology :: Sanger dideoxy sequencing
            ##Assembly-Data-END##
FEATURES             Location/Qualifiers
     source          1..1008
                     /organism="Herichthys tepehua"
                     /organelle="mitochondrion"
                     /mol_type="genomic DNA"
                     /db_xref="taxon:1830337"
                     /haplotype="F03F"
     CDS             <1..>1008
                     /codon_start=1
                     /transl_table=2
                     /product="cytochrome b"
                     /protein_id="QCY54448"
                     /translation="VWWNFGSLLGLCLAAQILTGLFLAMHYTSDIATAFSSVAHICRD
